# Comparison of the effects of ibuprofen and acetaminophen on PGE_2_ levels in the GCF during orthodontic tooth movement: a human study

**DOI:** 10.1186/2196-1042-14-6

**Published:** 2013-05-17

**Authors:** Niveditha Shetty, Anand K Patil, Sanjay V Ganeshkar, Srinidhi Hegde

**Affiliations:** 1 Department of Orthodontics, A.J. Institute of Dental Sciences, Mangalore, Karnataka 575004, India; 2 Department of Orthodontics and Dentofacial Orthopedics, S.D.M College of Dental Sciences and Hospital, Dharwad, Karnataka 580009, India; 3 Department of Conservative Dentistry and Endodontics, A.J. Institute of Dental Sciences, Mangalore, Karnataka 575004, India

## Abstract

**Background:**

Pain is among the most cited negative effects of orthodontic treatment. Non-steroidal anti-inflammatory drugs seem to be an effective option for minimizing this but can have adverse effects on tooth movement owing to their ability to block prostaglandin synthesis. Acetaminophen has been suggested as the analgesic of choice during orthodontic treatment as it showed no effect on orthodontic tooth movement in previous animal studies. The purpose of this study was to compare the effects of ibuprofen and acetaminophen on the prostaglandin E_2_ (PGE_2_) levels of the gingival crevicular fluid (GCF) during orthodontic tooth movement in human subjects.

**Methods:**

A total of 42 patients (mean age 18 ± 4.5 years) were randomly divided into three equal groups: ibuprofen, acetaminophen, and control groups. Maxillary canines were distalized with 150 g of force delivered by NiTi coil springs. GCF samples were obtained before (baseline) and after spring activation at 24, 48, and 168 h. The PGE_2_ content of the GCF was determined using enzyme-linked immunosorbent assay.

**Results:**

PGE_2_ levels in all groups increased significantly by 24 and 48 h of force application and decreased to baseline levels by 168 h. No significant difference was found between the acetaminophen and control groups at any time point. There was a significant decrease in PGE_2_ levels in the ibuprofen group at 24 and 48 h when compared to the other two groups.

**Conclusions:**

Acetaminophen showed no significant effect on prostaglandin synthesis and may be the safe choice compared to ibuprofen for relieving pain associated with orthodontic tooth movement.

## Background

Orthodontic tooth movement is known to cause inflammatory reactions in the periodontium and dental pulp, which stimulate release of various biochemical mediators. This is often associated with painful reactions, which have been rated as the greatest dislike during and fourth among major fears prior to orthodontic treatment [1]. Prostaglandins, particularly prostaglandin E_2_ (PGE_2_), have been implicated as one of the main mediators of this inflammatory reaction which increase the vascular dilatation and permeability and induce bone resorption through osteoclastic cell activation [2]. Evidence indicates that the local application of prostaglandins in the form of subperiosteal injections resulted in increased tooth movement in both rats and humans [3-8]. A reflection of the inflammatory process during orthodontic tooth movement can be observed as elevated concentrations of chemical mediators in the gingival crevicular fluid (GCF) of the moving teeth. Nowadays, this is commonly used as a biomarker assay to assess the level of various chemical mediators such as prostaglandins and indicators of root resorption such as dentin degradation products [9].

Non-steroidal anti-inflammatory drugs (NSAIDs), which inhibit cyclooxygenase activity thereby affecting the synthesis of prostaglandins, remain the most preferred method for pain control during orthodontics [10,11]. Acetaminophen, a non-steroidal anti-inflammatory drug in the family of para-aminophenols, said to have a central analgesic effect, showed no effect on orthodontic tooth movement in previous animal studies [12-14]. However, human studies in this regard, which evaluate the direct effect of acetaminophen on the production of prostaglandins and thereby on orthodontic tooth movement, are lacking. It is debatable whether findings from animal experiments can be extrapolated to the human situation as morphological and physiological differences between animal and human alveolar bone and periodontal ligament have to be considered [15]. Moreover, acetaminophen has been shown to either inhibit or stimulate prostaglandin synthesis, depending on the tissue, preparation of the tissue, and constituents of the incubation milieu [12,16,17]. It is also known to reduce the levels of prostacyclins after systemic administration in humans [18].

The aim of the present study was to compare the effects of ibuprofen and acetaminophen on the PGE_2_ levels in the GCF during orthodontic tooth movement in human subjects. By studying the alterations in the levels of these mediators, the possible effects of these drugs on the biologic processes mediating orthodontic tooth movement in humans may be evaluated.

## Methods

Study subjects consisted of 42 patients (mean age 18 ± 4.5 years) seeking orthodontic treatment, in whom bilateral maxillary first premolar extraction was planned. They were randomly divided into three equal groups of 14 subjects each. The first group was prescribed with ibuprofen, the second with acetaminophen, and the third group was taken as the control group, without administration of any drug. All patients were checked for periodontal status, and those with a history of systemic diseases, gastric disorders, or history of intake of any medication within the past 6 months were excluded from the study. The research was carried out in compliance with the Declaration of Helsinki after obtaining ethical approval from the Institutional Review Board, SDM College of Dental Sciences and Hospital, Dharwad, India.

Fixed orthodontic therapy was started on all patients after obtaining informed consent. All the patients were on strict mechanical oral hygiene regimen. After initial leveling and aligning, the maxillary canines were retracted on a 0.018-in. stainless steel wire with 150 g of force delivered by nickel titanium tension springs (Orthoforce G4-Nickel Titanium, G&H Wire Company, Hanover, Germany) placed between the maxillary molars and canines. The subjects received a Nance button for anchorage control. At the appliance activation, the subjects in the first group were given ibuprofen, 400 mg three times daily for 2 days. The second group received acetaminophen, 500 mg three times a day for 2 days. The third group did not receive any analgesics.

**Figure 1:**
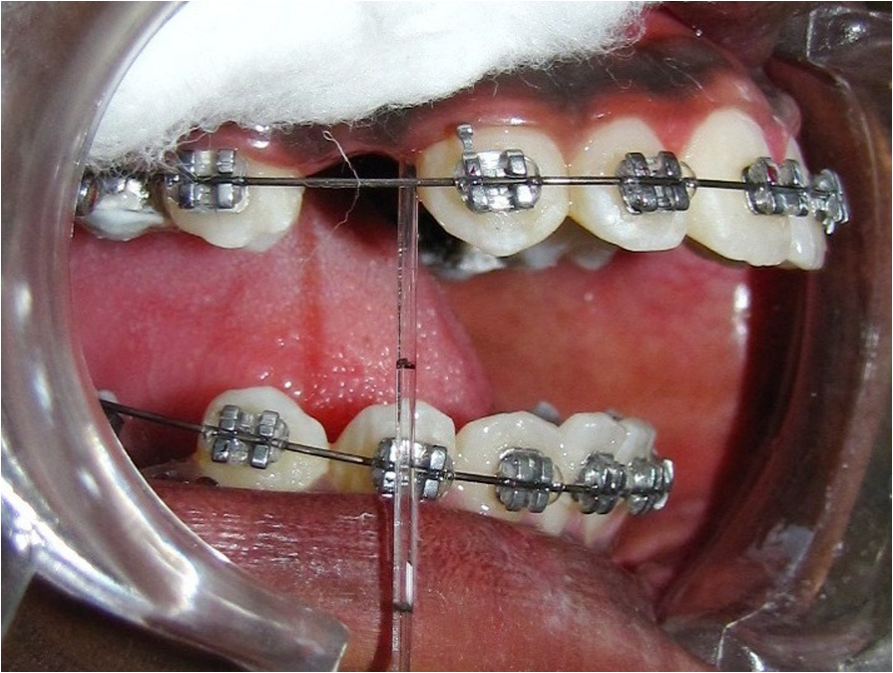
**GCF collection using micropipettes at baseline (*T***_**0**_**).**

GCF sampling was done before the placement of the closed coil springs (baseline *T*_0_) and after the activation of the springs at 24 (*T*_1_), 48 (*T*_2_), and 168 (*T*_3_) h. Each sample of GCF was collected from the gingival crevicular sulcus of the maxillary canine using calibrated micropipettes by capillary action (Figure 1). The area was isolated using cotton rolls to prevent saliva contamination, and GCF was collected by placing the microcapillary pipettes at the entrance of the gingival sulcus, gently touching the marginal gingiva. From each test site, a standardized volume of 2 μl was collected using the calibration on white color-coded 1 to 5 μl calibrated volumetric microcapillary pipettes (Sigma-Aldrich Chemical Company, St. Louis, MO, USA) using an extracrevicular approach (‘unstimulated’). Each sample collection was allotted a maximum of 30 min, and some test sites that did not express any volume of GCF within the allotted time were excluded from the study. The samples were diluted in phosphate buffer solution and stored at −20°C. Once all the samples were obtained, immunoassay for PGE_2_ was performed. The quantitative PGE_2_ content of the crevicular fluid was determined using the Neogen prostaglandin E_2_ enzyme-linked immunosorbent assay kit (Neogen Corporation, Lexington, KY, USA), according to the manufacturer's instructions.

The sample and standard solutions were first added to the antibody precoated microplate. Next, the diluted enzyme conjugate was added, and the mixture was shaken and incubated at room temperature for 1 h. The plate was then washed removing all the unbound material. The bound enzyme conjugate was detected by the addition of substrate which generated an optimal color after 30 min. Hydrochloric acid (1 N) was added to each well to stop the enzyme reaction. The plate was read using a 450-nm microplate reader. Quantification of PGE_2_ in the samples was achieved by comparison with a standard curve generated from known quantities of PGE_2_ (standards) that had gone through the assay.

### Statistical analysis

Friedman test was performed to determine if statistically significant differences were present between the four different time points in the three groups. Bonferroni correction was applied after which within-group differences of the PGE_2_ levels in the gingival crevicular fluid between 0 and 168 h were evaluated by the Wilcoxon matched-pairs test.

Differences of the PGE_2_ levels at different time periods between the three groups were determined by the Kruskal-Wallis test. Pairwise comparisons of the groups were done using the Mann-Whitney *U* test.

## Results

### Intragroup differences

The output of the Friedman test showed the presence of statistically significant differences (*p* < 0.05) between the four time points in all the three groups. Prostaglandin E_2_ levels in the GCF significantly increased in all groups by 24 h when compared to baseline values (*p* = 0.001). The PGE_2_ levels at 48 h in the three groups were also significantly high compared with baseline values (*p* = 0.001). Prostaglandin E_2_ levels at 168 h were not significantly different from baseline values (Table 1). Prostaglandin E_2_ levels of GCF decreased significantly in all the groups between 24 and 48 h, between 24 and 168 h, and between 48 and 168 h (*p* = 0.001, Table 1).

**Table 1:** **Intragroup and intergroup comparisons of PGE**_**2 **_**levels (ng/mL)**

	***T***_**0**_	***T***_**1**_	***T***_**2**_	***T***_**3**_	**PGE**_**2 **_**levels ( *p *)**					
					***T***_**0 **_**vs. *T***_**1**_	***T***_**1 **_**vs. *T***_**2**_	***T***_**2 **_**vs. *T***_**3**_	***T***_**0 **_**vs. *T***_**2**_	***T***_**0 **_**vs. *T***_**3**_	***T***_**1 **_**vs. *T***_**3**_
Intragroup										
Group I	31.90 ± 8.88	47.94 ± 10.28	36.99 ± 8.80	32.35 ± 8.96	0.001	0.001	0.001	0.001	0.463	0.001
Group A	32.85 ± 9.09	63.00 ± 9.08	47.70 ± 9.24	33.01 ± 10.42	0.001	0.001	0.001	0.001	0.875	0.001
Group C	34.15 ± 13.59	67.15 ± 16.79	51.60 ± 16.65	35.04 ± 13.83	0.001	0.001	0.001	0.001	0.753	0.001
Intergroup (*p*)										
I vs.A	0.963	0.0006	0.011	0.490						
I vs.C	0.963	0.002	0.011	0.679						
A vs.C	0.747	0.421	0.520	0.963						

*T*_0_ = baseline, *T*_1_ = 24 h, *T*_2_ = 48 h, *T*_3_ = 168 h. I, ibuprofen group; A, acetaminophen group; C, control group.

### Intergroup differences

The PGE_2_ levels of the ibuprofen group at 24 h were significantly different when compared to the acetaminophen and control groups (*p* = 0.0006 and *p* = 0.002, respectively, in Table 1). At 48 h, the PGE_2_ levels in the ibuprofen group showed statistically significant differences when compared to the acetaminophen and control groups (*p* = 0.011, Table 1). No significant differences in PGE_2_ levels were found between the acetaminophen and control groups at any time measured (Table 1). No significant difference was found between the three groups at baseline and 168 h (Table 1).

## Discussion

The early phase of orthodontic tooth movement is characterized by inflammatory responses of the periodontal tissues with osteoblastic and osteoclastic remodeling. Reported patient discomfort as well as pain is generally at its highest during the first 24 h after the application of an orthodontic force. The periodicity of these complaints peaks at 24 h and decreases to baseline levels by 7 days [19,20]. NSAID usage is the most routinely used pain management method during orthodontic therapy.

The present study analyzed with the use of GCF the effects of two popularly used analgesics, ibuprofen and acetaminophen, on PGE_2_ levels. While high doses of NSAIDs have been reported to disrupt tooth movement in previous animal studies [12,21,22], the possible effects of commonly used analgesics in over-the-counter doses on the biologic processes underlying tooth movement have not been evaluated. We have tried to evaluate the effects of ibuprofen and acetaminophen in over-the-counter doses in our study.

Quantitative evaluation of PGE_2_ from GCF samples of human subjects in our study showed that the PGE_2_ levels in all the experimental groups increased significantly by 24 h and maintained the elevated levels until 48 h of orthodontic force application when compared with baseline measurements (*p* = 0.001). We observed a statistically significant decrease in the level of inflammatory mediators as time progressed which is evident in the periodical evaluation at 48 and 168 h. The gradual suppression of the inflammatory reaction and the decay in orthodontic force most likely account for this decrease in levels of PGE_2_. Our findings are in concordance with the studies of Grieve et al. [23] and Sari et al. [2] who also quantified GCF PGE levels after 24 and 48 h of appliance activation and found significant elevations when compared with the baseline values. Lee et al. [24] also found an increase in PGE_2_ levels in the GCF after 24 h of orthodontic force application. The clinically undetectable gingival inflammation usually caused during fixed orthodontic appliance therapy [25,26] might have contributed to the increase in PGE_2_ levels at 24 and 48 h of appliance activation.

In our study, the control group received no pharmacological agent; therefore, it is assumed that the mean concentrations of PGE_2_ released in the GCF were unaltered and purely the result of orthodontic force application. The samples of the acetaminophen group showed some decrease in the PGE_2_ levels when compared to the control group, but the difference was not statistically significant. No statistically significant difference was observed between the acetaminophen and control groups at any time point, indicating that PGE_2_ levels were not affected significantly by acetaminophen administration. This finding supports the theory that acetaminophen fails to exhibit peripheral anti-inflammatory activity, i.e., it does not block prostaglandins peripherally because it does not concentrate in areas of inflammation where the peroxide level is high [18,27,28]. The explanation for this could also be that NSAIDs block COX-1 and/or COX-2, whereas paracetamol blocks a third isoform, COX-3, which is expressed only in the brain and spinal cord and therefore has minimal effects on prostaglandin synthesis [29-32].

We found a statistically significant decrease in PGE_2_ levels in the ibuprofen group at 24 h (*p* = 0.002) and 48 h (*p* = 0.011) when compared to the control group. There was also a highly significant difference when comparing the mean concentrations of PGE_2_ between the two drug groups at 24 h (*p* = 0.006) and 48 h (*p* = 0.011). This indicated that ibuprofen inhibits PG synthesis more than acetaminophen during the first and second days of orthodontic tooth movement. This result was similar to that of Kehoe et al. [13], who found a significant decrease in the mean concentration of PGE_2_ levels in the PDL exudates of guinea pigs in whom ibuprofen had been administered. This implies that by inhibiting prostaglandins, ibuprofen may have an effect on the rate of orthodontic tooth movement [16,21,22,33]. The highly significant decrease in PGE_2_ levels in the ibuprofen group when compared to the other two groups might be attributed to its anti-inflammatory action on peripheral inflamed tissues.

In our study, we have evaluated the effects of two analgesics, ibuprofen and acetaminophen, on the PGE_2_ levels in the GCF during orthodontic tooth movement. Although it is implied that NSAIDs like ibuprofen may impede tooth movement by inhibiting PG synthesis, the rate of tooth movement in the groups has not been measured. Long-term comparison of the rate of tooth movement in patients with administration of these drugs would yield definitive results. No evaluation of the efficacy of the two drugs prescribed in relieving pain associated with appliance activation was done in our study. However, comparison of the efficacy of ibuprofen and acetaminophen in controlling pain after orthodontic tooth movement in a previous study [34] has shown that both are equally effective in reducing discomfort after initial orthodontic appliance placement. In another study, acetaminophen group showed visual analogue scale results similar to those of naproxen sodium and aspirin [20]. Therefore, acetaminophen maybe deemed to be as effective as ibuprofen, naproxen sodium, or aspirin in alleviating pain following appliance placement.

## Conclusions

The following conclusions were drawn from this study:

1. The PGE_2_ levels of the three groups peaked at 24 h and decreased nearly to baseline levels by 168 h. Therefore, the pain suppressant drugs prescribed in this period could adversely affect the PGE_2_ synthesis and hence the rate of orthodontic tooth movement.

2. Ibuprofen inhibited PGE_2_ synthesis significantly more than acetaminophen and when compared to the control group.

3. Acetaminophen was not found to affect PGE_2_ levels significantly during the experimental period.

The results of this study suggest that NSAIDs like ibuprofen have an inhibitory effect on the release of prostaglandins during initial tooth movement and thereby may cause an impediment in the rate of tooth movement. Acetaminophen may be suggested as the drug of choice for safe and effective management of orthodontic pain.

## Competing interests

The authors declare that they have no competing interests.

## Authors’ contributions

The work presented here was carried out in collaboration among all authors. NS and AKP defined the research theme and designed the methods and experiments. NS carried out the GCF collection and laboratory assay, analyzed the data, interpreted the results, and drafted the manuscript. SH worked on the data interpretation. SH and SVG discussed the analyses, interpretation, and presentation of data. All authors read and approved the final manuscript.
